# Real-world experience of OnabotulinumtoxinA treatment in female patients with chronic migraine: a qualitative study using in-depth interviews

**DOI:** 10.1080/07853890.2023.2255215

**Published:** 2023-09-14

**Authors:** Domingo Palacios-Ceña, Carlos Ordás-Bandera, Javier Casas-Limón, Jorge Pérez-Corrales, Javier Güeita-Rodríguez, José A. Arias-Navalón, María-Luz Cuadrado

**Affiliations:** a Research Group of Humanities and Qualitative Research in Health Science of Universidad Rey Juan Carlos (Hum&QRinHS), Department of Physical Therapy, Occupational Therapy, Physical Medicine and Rehabilitation, Universidad Rey Juan Carlos, Alcorcón, Spain; b Department of Neurology, Hospital Universitario Rey Juan Carlos, Móstoles, Spain; c Department of Neurology, Hospital Universitario Fundación Alcorcón, Alcorcón, Spain; d School of Medicine, Universidad CEU San Pablo, Madrid, Spain; e Department of Neurology, Hospital Clínico San Carlos, Department of Medicine, Universidad Complutense de Madrid, Madrid, Spain

**Keywords:** (MeSH), botulinum toxin type A, drug therapy, migraine disorders, women, qualitative research

## Abstract

**Background:**

Chronic migraine (CM) causes great disability and affects an individual’s quality of life. OnabotulinumtoxinA (OBT-A, Botox®) was the first prophylactic treatment specifically indicated for CM. The aim of this study was to describe the experiences of women with CM treated with OBT-A.

**Materials and Methods:**

The study design is a qualitative descriptive study. A purposeful sampling of 30 women (mean age, 42.7; standard deviation, 10.6) who had received at least two administrations of OBT-A for CM (PREEMPT protocol) was performed. Data collection included in-depth interviews and researchers’ field notes. A thematic analysis was carried out according to qualitative research guidelines.

**Results:**

Five themes were identified: (a) A long way to go before Botox®, (b) First time hearing about the treatment and its expectations, (c) The administration of Botox®, (d) Treatment effects, and (e) Follow-up. Patients described a long history of treatment failures prior to the start of OBT-A treatment. Information about this migraine treatment came from the neurologist; following the information, patients had high expectations, including unrealistic expectations regarding the onset and duration of effect. They acknowledged fear of the injections and some discomfort due to the procedure. With treatment, participants reported better migraine control and an improvement in their quality of life. Follow-up had some barriers, such as delayed appointments for subsequent doses, but also strengths, such as effectiveness and few side effects.

**Conclusions:**

Qualitative research offers insight into how patients with CM experience treatment with OBT-A. Our results highlight some relevant aspects that should be considered when providing OBT-A treatment.

## KEY MESSAGES

Women had unrealistic expectations regarding the onset and duration of OBT-A effect.

With OBT-A treatment, women perceived better migraine control and improved quality of life.

Follow-up had barriers, such as delayed appointments for subsequent doses.

## Introduction

Chronic migraine (CM) is characterized by the presence of a headache for a total of more than 15 days per month for at least three months, with migraine characteristics present at least eight days per month [1]. CM overall prevalence is around 2% being the leading cause of chronic daily headache [2]. About 3% of patients with episodic migraine progress to CM each year [3,4]. Risk factors for CM are women, high baseline headache frequency, depression, high-frequency use or overuse of acute pain medications, and low socioeconomic status [2,5,6]. Various studies have shown that CM causes more disability and a greater impact in quality of life than episodic migraine [7–9]. It is also associated with more medical comorbidities, higher levels of anxiety and depression, more absenteeism from studies or from work and greater use of health care resources [10,11].

Preventive pharmacological treatment of CM is initially the same as for frequent episodic migraine. However, few treatments have demonstrated efficacy and safety specifically in CM in phase III clinical trials, namely topiramate [12,13], onabotulinumtoxinA (OBT-A, Botox®) [14,15] and monoclonal antibodies to calcitonin gene-related peptide or its receptor [16–19]. OBT-A was the first prophylactic treatment specifically indicated for CM, following the publication of the pivotal randomized controlled trials PREEMPT 1 and PREEMPT 2 [14,15]. Since its approval for CM, the effectiveness, safety, and tolerability of OBT-A has been confirmed in long-term open-label prospective studies (such as COMPEL and FORWARD) as well as in many real-world observational studies [20–22]. Together, these studies have shown that treatment with OBT-A reduces the frequency of headache and acute headache pain medication intake per month. They have also shown that treatment with OBT-A decreases disability and improves patients’ quality of life, as measured by standardized scales [20]. However, for pain syndromes (such as migraine), other non-quantifiable aspects must be considered [23].

Qualitative research provides a tool for obtaining real-world data from patients that complements and enriches the data obtained through quantitative research [24,25]. It is particularly beneficial for describing complex phenomena such as individual health-related behaviors, considering their beliefs, values, and motivations [26,27]. Qualitative studies have already been used to analyze patients’ experiences with OBT-A therapy in other neurological disorders such as poststroke spasticity [28–30], cerebral palsy [31,32], oromandibular dystonia [33], and spasmodic dysphonia [34]. In contrast, the evidence regarding female patients’ experiences with CM on treatment with OBT-A is scarce. Recently, Wilderman et al. [35] explored the experience of a mixed and heterogeneous group of patients with prophylactic OBT-A treatment for CM including both male and female participants. Accordingly, the aim of this study was to describe the experiences of women with CM treated with OBT-A.

## Material and methods

### Study design

A qualitative descriptive and exploratory study was conducted based on an interpretive framework [36–39], following the Standards for Reporting Qualitative Research (SRQR) [40] and the Consolidated Criteria for Reporting Qualitative Research (COREQ) [41]. This study design stays close to the individual’s own words when the participants describe their experiences, aiming to describe ‘what and how is happening’, which leads to a rich description of the phenomenon of interest [37,42,43]. It also seeks to be a comprehensive summary of events in the everyday terms of the described event [37,38]. The study protocol was approved by Fundación Jiménez Díaz (code: PIC144-19_HRJC) and Hospital Universitario Fundación Alcorcón (code: 19/100) ethical committees. Written and verbal informed consent was obtained prior to the participant’s inclusion in the study. Also, the present study adhered to the Helsinki Declaration.

### Participants, context, and sampling strategies

A non-probabilistic, purposeful sampling strategy was used based on relevance to the research question rather than representativeness [26,44]. This sampling strategy selects participants deliberately [26].

The inclusion criteria were: (a) Female sex, (b) Age ≥18 years and ≤65 years, (c) Confirmed diagnosis of CM, based on the criteria of the 3rd edition of the International Classification of Headache Disorders [1], prior to treatment with OBT-A (Botox®), (d) Treatment with OBT-A in accordance with the PREEMPT protocol (155-195 U) [45] with two or more sessions of Botox® injections, and (e) Sufficient capacity to understand and approve the informed consent form. The exclusion criteria consisted of: (a) Coexistence of other primary or secondary headaches, (b) Coexistence of other chronic pain syndromes, (c) Severe psychiatric illness, and (d) Concomitant severe systemic disease.

Patients were selected directly from those on OBT-A therapy for CM in the Headache Units of the Neurology Departments at Hospital Universitario Rey Juan Carlos (Móstoles, Madrid) and Hospital Universitario Fundación Alcorcón (Alcorcón, Madrid).

The sample size estimation was guided by the proposal of Turner-Bowker et al. [46], who reported that around 30 interviews is needed to emerge 99.3% of concepts, themes, and content. Forty-three participants who met the inclusion criteria were recruited. Of these, one withdrew from the study due to personal problems, two changed their minds, and ten participants did not recontact or respond to the researchers’ attempts to contact them.

### Data collection

The collection of the core data for this study was based on in-depth interviews and the researcher’s field notes [26,44]. During the first stage (participants 1–10), we used unstructured interviews with open questions [44] such as: ‘What is your experience of Botox® treatment? What is the most significant thing about your illness and Botox® therapy?’ During the interview, the researchers captured key words and topics and retrieved them on related questions to clarify the content [26]. The new areas emerged from the first stage, that ended in the 10th interview, required further exploration, which led to the second stage (participants 11–30). The second stage was composed by semi-structured interviews that were designed to address specific topics of interest, which are described in Table 1 [26]. This question guide was constructed based on the interviews from the first stage, not based on the perspective or interests of the researchers. Open-ended follow-up questions were also used to obtain detailed descriptions. Additionally, the expression ‘Please, tell me about that’ was used (if necessary) to increase the depth of the discussions surrounding specific topics in each interview.

**Table 1.** Semi-structured interview guide.

Research areas	Questions
Botox® and prior treatments for migraine	What is the most significant thing for you about the treatment prescribed prior to the use of Botox®? What is the most significant thing about the Botox® treatment? Can you tell us about your experience before and after the Botox® treatment? Regarding the treatment previously prescribed, what barriers or facilitators influenced whether or not you continued with it? Regarding Botox® therapy, what barriers or facilitators influenced whether or not you continued with it? What are the factors that helped you to have confidence in the treatment?
Relationship with the treating physician	What role does the doctor play regarding Botox® therapy? What do you consider to be the most important factors in the therapeutic relationship?
Daily life, personal relationships, and work environment	What have been the most significant changes in your daily life following the treatment with Botox®? How has it affected your personal relationships with your friends, family, and work colleagues?

During the COVID-19 pandemic, face-to-face interviews were not possible, so online interviews were performed using *Microsoft Teams* [47], which allowed audio and video record. A total of 1392 min of interviews were recorded (mean 46.4 and standard deviation [SD] 11.5 min per interview). The interviews were conducted by DPC, JPC, and JGR, who complemented the information (as a secondary source) with their field notes. Relevant demographic and clinical data were collected by COB and JCL from the medical records, including age, migraine history, treatments used, duration and dose of OBT-A treatment, frequency of headaches before treatment, current frequency of headaches and side effects of OBT-A treatment.

### Data analysis

Both in-depth interviews and the researcher’s field note were fully transcribed. A thematic analysis was carried out on these texts by identifying the text fragments that could provide relevant information according to the research question [44]. Firstly, codes were identified as the most descriptive contents. Subsequently, the codes were grouped in categories according to their content’s similarity [44,48]. Then, the categories were grouped as themes, considering common contents that could describe the participants’ experience [49]. Two thematic analyses were performed separately by JPC and JGR. Then, results were combined through joint team meetings, where final themes were displayed, combined, integrated, and identified. In case of divergence of opinions, the team discussed to reach a consensus. No qualitative software was applied for the data analysis.

### Rigor

Table 2 describes the strategies to control trustworthiness [50].

**Table 2.** Trustworthiness criteria.

Criteria	Techniques performed and application procedures
Credibility	Investigator triangulation: two researchers analyzed each interview. Thereafter, team meetings were performed in which the analyses were compared, and themes were identified.
	Triangulation of data collection methods: unstructured, and semi-structured interviews were conducted, and researcher field notes were kept.
	Member checking: the participants were asked to confirm the data obtained. All participants were offered the opportunity to review the audio and/or video records to confirm their experience. None of the participants made additional comments.
Transferability	In-depth descriptions of the study were performed, providing details of the characteristics of researchers, participants, contexts, sampling strategies, and the data collection and analysis procedures.
Dependability	Audit by an external researcher: an external researcher assessed the study research protocol, focusing on aspects concerning the methods applied and study design.
Confirmability	Investigator triangulation, and data collection triangulation. Researcher reflexivity was encouraged via the completion of reflexive reports and by describing the rationale for the study.

## Results

Thirty female patients were finally interviewed, with a mean age of 42.7 years (SD 10.6); the mean time since the onset of migraine was 23.8 years (SD 10), and the median time since migraine had become chronic was 7.1 years (interquartile range 5–9.9 years). All the participants had tried between 2 and 9 oral migraine preventives, with no response or poor response to the treatment. Time on OBT-A treatment ranged from 5 to 147 months, with a mean of 49.6 months (SD 28.7). The mean headache frequency was 21 days per month (SD 5.6) before the start of OBT-A treatment, and 6.1 (SD 5.2) in the last three months. Twenty-six patients (86.7%) had achieved at least a 50% reduction in headache frequency. Side effects of Botox® treatment were only recorded in three patients (P8, P12, P17), consisting of cosmetic changes. Demographic and clinical data of each participant are shown in Supplementary Additional file 1.

Five specific themes with its categories were identified: (a) A long way to go before Botox®, (b) First time hearing about the treatment and its expectations, (c) The administration of Botox®, (d) Treatment effects, and (e) Follow-up. Table 3 reproduces some of the participants’ responses, taken directly from the interviews, to illustrate the themes.

**Table 3.** Narratives of participants.

Theme	Narratives
**A long way to go before Botox®**	**Long history of pharmacological treatments:** * ‘I have been suffering from headaches since I was 19 years old, and I am now 63 (…), they have given me all the medicines they could give me, all the medicines on the market…’. (P20, 63 years old-yo)* **Trial and error:** * ‘I have spent many years trying everything, changing medication because it stopped working (…), it didn’t let me move forward. Always, if it wasn’t one thing, it was another. It was trial and error’. (P1, 46 yo)* **Side effects:** * ‘Some of the pills make me sleepy (…), if I’m active or on the go, nothing happens, but if I stop, it knocks me straight out. I feel sluggish, my reflexes are slower (…) I don’t perceive everything in the same way, everything moves as if in slow motion’. (P11, 41 yo)* **Change and abuse of medication:** * ‘Nolotil is like having a sweet (…). Nolotil does nothing for me, I can take 4 every two hours and nothing. One day I took 5 or 6 ibuprofen in a row, and it didn’t relieve the pain’. (P4, 36 yo) ‘I do what the doctor tells me to do…if it works. If not, I start experimenting and mixing medication, until something does work’. (P8, 37 yo)* **Going to the emergency department:** * ‘When I couldn’t take it any more, I would go to the emergency department, and they would give me medication straight into my vein until they got the pain under control. They’d stop the headache, and then I’d go home again! Until the next one’. (P1, 41 yo)* **Starting directly with Botox®:** *’If they know Botox works so well, why do we have to take so many drugs before they put us on something that has been proven to work better? Why is there so much trouble getting access to Botox? Why have they let us suffer for so long?’. (P24, 23 yo)*
**First time hearing about the treatment and its expectations**	**Information gaps, fears, and false beliefs:** * ‘That’s more than 30 injections in the head! I could only think of how deep the needle in the head would go… That’s why I was so reluctant. But they did a good job of explaining it to me. I didn’t know it was so superficial’. (P19, 50 yo) ‘It should be explained differently because people are reluctant. You hear Botox, and you assume that they paralyze you, that your face will be paralyzed, that they will jab you… When it comes to explaining it, they only focus on aesthetics, and it is NOT just about aesthetics. When I tell people that I have it done, they laugh’. (P21, 46 yo)* **Quality of the information provided by the neurologist:** * ‘They explained it to me. The area where they would inject: the forehead, the head, the number of injections (…) It is necessary for them to explain to you both the good and the bad. They were honest in telling me that there were people to whom it did absolutely nothing’. (P11, 41 yo)* **Trust:** * ‘You always trust the person you’ve been with for years more. The first time you are injected comes as a bit of a shock because it’s a bit scary, but because I’ve been with him for so many years, I trust him completely. If someone else did it, I would be more tense: not knowing them, I wouldn’t trust them at all’. (P2, 41 yo)* **Consistency and concern:** * ‘It is very important to me that the doctor asks me how I feel, and how I am, both before or after. To feel that he is keeping a close eye on me’. (P10, 40 yo)* **Having time:** * ‘It’s all done in a very short time, sometimes the doctors are more detached, as if they were in a hurry (…) I hate needles, but when you feel listened to and when they explain things calmly, even if it has been a short time, and I don’t feel they are in a hurry, that gives me peace of mind’. (P22, 46 yo)* **Personalized treatment:** * ‘… that they offer you alternatives. By saying to me: <which side hurts more, the right or the left?> That seems fundamental to me, for them not to work in automatic. I am not just another patient. I’d like the Botox injections to be a bit personalized’. (P28, 34 yo)* **High expectations for the pain to go away:** * ‘The neurologist where I was going said < it will go away>, a lie. Your migraine won’t go away just because you get Botox. It will reduce the pain, but it won’t go away’. (P18, 23 yo)* **Delayed effect:** * ‘I was nervous for the first few days, waiting for it to take effect. But I had to wait a week or so before I noticed anything’. (P15, 23yo) ‘At first, I noticed a little change, but not much; the migraines continued every day. After about the sixth or eighth month, everything started to change’. (P10, 40 yo)* **Duration of the effect:** * ‘After the third month, the pain starts to return, more continuous, more daily, but it’s only discomfort. I don’t get the strong migraines I used to get before (…)’. (P10, 40 yo)* **Emotion curve during the waiting period: ** *‘At first, it didn’t really work for me. The first month was a downer because I went in with high expectations, and it wasn’t very effective, but as the months went by I got better and better. It was a rollercoaster of emotions’. (P16, 42 yo)*
**The administration**	**Fear:** * ‘The first time they infiltrate you, it’s shocking, it’s scary. It hurts a little, but you can bear it. But seeing the needle getting closer… I’d just close my eyes’. (P2, 41 yo)* **The jab and the discomfort: ** *‘I always put up with the jab because I know it is going to be for a long-term improvement. But when the needle goes in and comes out, and I feel how it is being put in, it is very unpleasant’ .(P24, 23 yo) ‘It hurts because it does, but it is a pain that can be tolerated perfectly. The thing is that as you see the needle coming closer, and as they start injecting you with it, it is a bit unpleasant, but with all the benefit that it gives you, it’s wonderful’. (P27, 55 yo)* **First days with discomfort:** * ‘It leaves me with quite a headache for 4 or 5 days (…) there is intense pain in the areas where you have been injected. Then it goes away, but you do feel off for the first 3 or 4 days after the injection’. (P2, 41 yo)* **A small price to pay:** * ‘… You spend a week with a sore head, but it’s a small price to pay for two and a half months of quality of life’. (P25, 42 yo) ‘The jabs hurt, but if it weren’t for Botox I wouldn’t be able to have a life, I would be a person with a disability’. (P30, 50 yo)*
**Treatment effects**	**Reduced pain frequency and intensity: ***‘It has changed my life. It has reduced the pain in duration, and in intensity. If I used to have 30 episodes, now I have 2 or 3 a month. It is remarkable (…). It is bearable pain, which was not the case before. It reduces the pain, the intensity, the episodes. It reduces everything.’ (P5, 45 yo)* **Reduced medication usage: ***‘Botox has reduced the number of migraines, but the most significant thing is that it has removed that aura of pain that I always had every morning when I woke up (…) and it is true that I have to take less medication, I recover faster’. (P2, 41 yo) ‘…life before Botox was to be afraid of your attacks. I used to carry my medication with me because it could hit you at any moment, you can’t live in peace, you’re afraid of it. Since I’ve had Botox, I don’t carry medication anymore, I don’t remember the last time I had to take anything’. (P21, 46 yo)* **Visits to the emergency department: ***‘I’m much better because I have fewer crises (…) it was overwhelming, I would be in the emergency room all day because of the pain. Since I started having Botox injected, I have only had to go to the emergency room a couple of times a month, more or less. Before it was practically 3 times a week’. (P11, 41 yo)* **Changes in appearance:***’There are aesthetic changes: a raised eyebrow, skin that turns red… But what is striking is the eyebrow being out of place…’. (P8, 37 yo)* **Improved quality of life: ***‘Before, I would always have to take medication every day and deal with the side effects that all migraine treatments have. Now, by being injected every 3 or 4 months, and the long-lasting effect it has, it has improved my overall quality of life’. (P2, 41 yo) ‘The main change is that I am happy, I want to do things, I can spend more time with my daughter’. (P22, 46 yo)* **Getting their lives back: ***‘For me, it’s my life. I cannot live without Botox, without it I would have no life (…) I’m super happy, my life is different, I’m a different person, it has given me a new life’. (P1, 46 yo) ‘Without Botox, I felt terrible, I had no life. With Botox, I feel much better and can live my normal life, do things that I did before (…) for me, it has been a lifesaver because it has saved me from my daily life of suffering’. (P20, 63 yo)* **Not feeling sick: ***‘The pain was exasperating. I felt very bad, very sick. I spent many, many years like that, until I got Botox injections. With them, I changed: my life has really changed, I don’t feel sick anymore’. (P26, 37 yo)* **Peace of mind: ***‘Now I feel completely at peace. Maybe sporadically I’ll have a migraine or two. It has given me a lot of peace of mind (…) not having to depend on pain and its limitation. I’m relieved. It’s taken a huge weight off my shoulders and now, thanks to the toxin, I can live better, I’m more complete, because I can do activities, a lot of things that I couldn’t do before’. (P10, 40 yo)* **Planning meetings and activities: ***‘I’m back to being myself, the way I am when I don’t have a headache. I can do everything, I can meet people, I can eat out, I can have meetings, I can meet my family. It’s like going back to my old life, to the way it was before the headaches’. (P20, 63 yo)* **Improved relationship with family:***’You notice the difference because you don’t have pain anymore, and you can interact more with your family, with your environment. From being in a locked room to being able to be with your family’. (P3, 29 yo) ‘Botox means being able to enjoy my whole family, to get out of my cave [room] and be able to celebrate my daughters’ birthdays, to continue having relationships or go out for dinner, (…) to be able to enjoy myself, without being afraid that if I have a headache, I will have to leave’. (P5, 45 yo)* **Improved in the couple relationship: ***‘I have noticed an improvement in my relationship with my partner. He told me that when I had a headache, it was like living with a piece of furniture. I didn’t do anything, I was just sitting at home, not moving. Now we can share moments and go out again’. (P4, 36 yo)* **Continue working: ***‘The treatment allows me to continue working and to stop taking sick leave. Without Botox, I would have no life’.* (P1, 46 yo) *‘The biggest impact is that I haven’t stopped working, studying. It allows me to live my life. That is the main way it has affected me, because before, even if I took tablets, there were days when the pain was impossible to control’. (P18, 23 yo)* **Improved performance: ***‘Workwise, it has meant a lot to me, because I have a very psychologically demanding job. If I am not well, my work will reflect this (…) I am much more efficient after the treatment because I can concentrate better, and my mood has also improved’. (P2, 41 yo) ‘It’s giving me good results. My performance is better, my concentration has improved, my mood is better. For me, it has all been positive’. (P28, 34 yo)*
**Follow-up**	**Delayed appointments: ** *‘It’s very important to schedule the next injection because there are many people who are being treated with Botox who can’t get an appointment in time and have to put up with the pain’. (P18, 23 yo)* **Difficulty in continuing treatment: ** *‘The only drawback that would make me switch to another treatment is the difficult access to treatment continuity. I don’t know the reasons, either because of the doctors or the management, but they can’t maintain the continuity for the treatment to be effective. It’s no use having two and a half good months if I’m going to have eight bad ones (…) There needs to be a regularity in the administration of the treatment’. (P23, 45 yo)* **Effect of the COVID-19 pandemic: ** *‘After COVID, the waiting lists were very long. They cancelled all the treatments. I waited about 7–8 months, I think I almost reached a year from one treatment session to the next. In that year, the relapse was remarkable (…) As time went by, my head hurt more and more’. (P19, 50 yo)* **Getting rid of the pain: ** *‘I like my doctor very much, he’s a lovely person, but I go because he gives me Botox and it works. Otherwise, they wouldn’t see hide nor hair of me’. (P4, 36 yo)* **Just one jab: ** *‘It’s easy to manage, you don’t have to take any medication, it’s easy, just a couple of jabs (…) Aesthetically, it’s noticeable, I have one eyebrow higher than the other, but I don’t care, as long as I’m not in pain’. (P12, 54 yo)* **Comfort: ** *‘This treatment is convenient for me, it only takes me 10 minutes to go to the hospital (…) Every four months, they email me to go to the hospital, and they inject me with it. All according to their schedule, and they monitor me perfectly’. (P21, 46 yo)*

### A long way to go before Botox®

Participants described a long history of drug treatments for their migraines prior to starting Botox® treatment. In most cases, these treatments were ineffective from the start or stopped working, and the patients experienced the successive prescriptions as a process of trial and error. In addition, the previously prescribed preventive drugs had caused many side effects, such as drowsiness, feelings of physical and cognitive slowing down, difficulties concentrating, memory loss or emotional lability. Other effects reported by participants included hormonal imbalances, tachycardia, dizziness, vomiting, dry mouth, change in taste and loss or gain of appetite, accompanied by weight loss or gain. During the years of migraine progression, acute pain medications were no longer able to control the pain. This caused many of the participants to start abusing the prescribed symptomatic medication (increasing doses) and/or to try different drugs at their own discretion. When the pain did not subside, going to an emergency department was the only way to control the pain. Having lived through many years of experiencing unresponsive treatment, many participants wondered whether it would not have been possible to start directly with Botox® and not delay the management of their pain, given that Botox® is a treatment with proven efficacy, with very good results.

### First time hearing about the treatment and its expectations

#### Information on and first time hearing about Botox®

The participants were not aware of the use of Botox® for migraine, and they first heard about it from their neurologist. Although many decided to accept the treatment in the absence of effective alternatives to manage their pain, being informed by the medical specialist helped, and in some cases precipitated their approval. In addition, the neurologist helped to fill information gaps and address fears and misconceptions that made them reject treatment *a priori*. Regarding the quality of information about Botox®, most participants agreed that the neurologist provided adequate information about the treatment, how it would be administered, the injection sites, the possible discomfort, and the realistic possibilities of pain relief. Factors that positively influenced their acceptance of Botox® treatment included: (a) Trust with the neurologist based on the time they had known the patient, (b) The doctor’s consistency and concern in asking, before and after the administration, about how the patient was feeling and if they were in any pain, (c) Having time for the patient and it wasn’t rushed (the faster they carried things out, the more aloof the doctor seemed), and (d) Offering alternatives to the injection site, since it meant that the care was more personalized and that the doctor did not work mechanically.

#### Waiting for the effects

Based on the feedback received, participants had high expectations for Botox® and most expected the pain to disappear soon and completely. However, some patients noted a delay in the onset of the effect lasting from several days to several weeks. There were cases who reported that the effects took several months, that they did not notice relief until the second or third injection, but eventually the pain subsided or decreased. Not seeing effects in the time expected caused frustration for some patients. On the other hand, some reported that their migraines did eventually return over the months following each injection, although usually at a lower frequency and intensity than before (often defined as ‘aches’). The patients described different emotional curves during the wait for the effects. First, they experienced a curve of hope, which corresponded to the high expectations placed on Botox® at the time of its administration; then a curve of frustration appeared, which corresponded to their disappointment during the wait and, finally, they ended up experiencing a curve of satisfaction, when they began to notice the effects of the treatment.

### The administration

The patients interviewed admitted that they were afraid on the first day of the Botox® injections because of the needles being inserted in the face and head. In addition, during the procedure they felt discomfort (due to the needle pricks and the injection of the liquid), although they admitted having tolerated it because they expected great benefits after the treatment. After the Botox® administration, the patients described feeling discomfort in the injection site and some even had a migraine for a few days. Gradually the symptoms were reversed: they felt increasingly less discomfort from the needle pricks until the headache diminished and/or disappeared. All participants agreed that, despite the discomfort, the injection was worth it (‘*it’s a small price to pay*’), as it eventually reduced their headache pain.

### Treatment effects

#### Perceived effects of Botox®

After the treatment, participants noticed a decrease in the frequency, duration, and intensity of their migraines. In addition, other preventive medications could be commonly withdrawn, and when symptomatic medication was required, the effect on pain reduction was greater than before Botox® treatment. Moreover, most of the participants (*n* = 23) reported that they had reduced their use of symptomatic (acute) pain medication. The number of times patients had to go to an emergency department for help also decreased, because they felt they could manage their pain on their own. During in-depth interviews, only three participants reported that Botox® injections could have aesthetic effects, such as changes in skin colouring after administration, or eyebrow elevation when the medication started to take effect.

#### Reclaiming their life

Participants described how the Botox® treatment had brought them joy and happiness, as they felt they were getting their life back and no longer felt sick. For them, not feeling sick meant reducing the constant dependence on medication and being able to regain continuity in their daily habits and activities. All participants regained quality of life, regained a fuller and more regular life, and had greater peace of mind. They also described how the Botox® treatment had positive effects on their relationship with their partner, family, and friends. They regained greater contact with them and returned to engaging in activities with them that they were previously unable to engage in. They felt that they had regained control of their lives by being able to plan activities and meeting people again, and by being able to share their time with their loved ones again without fear that pain would spoil everything. In addition, many participants described finding themselves again by being able to do things they could not do before. Many participants said that Botox® treatment had enabled them to resume their studies or return to work. They described how they had regained skills they had lost, as their migraines had decreased and were more manageable. They felt more productive at work/studies, concentrated better on their tasks, their mood had improved and felt they had another chance to continue with their academic or work life, regaining an important aspect of their life.

### Follow-up

Among the barriers that could negatively affect the continuity of Botox® treatment, patients identified: the management of appointments to receive and keep up with the course of the treatment, waiting lists to receive new doses of the treatment, and the lack of staff in the teams responsible for administering Botox®. The COVID-19 pandemic had a major impact on Botox® treatment: the cancellation of medical appointments and the postponement of outpatient treatments had an impact on the frequency with which patients received injections. On the patients’ side, in some cases the aesthetic effects (such as not being able to wrinkle their forehead or raised their eyebrows) or the uncertainty about the effect of the treatment were key factors.

According to the patients, one of the elements that facilitates adherence to treatment is the effectiveness of Botox® in managing migraines. They also pointed out the convenience of the treatment as a facilitating element: compared to taking daily pills, Botox® treatment means having to be injected only once every three months; in addition, the administration is quick and has few side effects.

## Discussion

Our results are consistent with previous qualitative studies [51–54] describing how CM causes a loss of autonomy and independence in patients and a sense of loss of control over their own lives. Before being treated with OBT-A, our patients lived with pain and its consequences for many years, without any effective treatments. In their qualitative study of the patients’ perspective on the use of OBT-A in CM, Wilderman et al. [35] also found that participants had tried a wide range of treatments previously, for many years, but the treatments did not help to reach a satisfactory level of function [55,56].

The REPOSE study [55], concerning real-life use of OBT-A in adults with CM, reported that only 10% of the 633 patients (*n* = 63/633) had previously received OBT-A as a migraine preventive; the remainder were OBT-A-naive. This delay could be partly explained by the statement in the European Headache Federation guidelines [57] that it is preferable for patients to have tried two or three other migraine prophylactics before starting with OBT-A. However, it has been shown that the likelihood of a positive outcome increases if treatment with OBT-A is started early, in the first 12 months after episodic migraine becomes CM [58]. It is therefore recommended that this treatment be started early [59]. Although treatment with OBT-A is generally suggested for CM patients with intolerance or lack of response to at least two oral preventive treatments, each case should be considered on an individual basis, taking into account the duration of CM, comorbidity with other diseases, and regular use of other treatments [57,60].

For our participants, the source of information about OBT-A was the neurologist, who informed them about the treatment, helped them understand it and answered all their questions. In the same vein, Wilderman et al. [35] described how patients were unsure how the treatment received could prevent CM, particularly because OBT-A is primarily known for its cosmetic effects.

Our participants had high expectations about the treatment’s potential to alleviate their pain. In Wilderman et al.’s study [35], patients who received OBT-A often had unrealistic expectations, ranging from very high expectations of recovery to no expectations at all. The expectations placed on treatment are influenced by what it means to be ‘effective’ to the patients. According to the same authors, treatment should be considered effective if it is able to reduce the frequency and/or severity of migraines, and if it allows patients to continue with their daily activities.

In addition, our participants described how not being able to reduce pain quickly after the first injection led to disappointment with the treatment, which disappeared over time as the efficacy of OBT-A became more and more apparent. All patients should be made aware in advance that the response to each administration of OBT-A may take a few days or weeks to appear (latency of effect). On the other hand, some of our patients reported not noticing an effect until the second or third injection. This is in line with Silberstein et al. [61], who reported that 49% of CM patients had a ≥ 50% reduction in headache-day frequency after the first OBT-A injection, but about 11% and 10% of patients first responded during cycles 2 and 3, respectively. In fact, it is recommended to treat with OBT-A at least a second and a third time before establishing non-response to this treatment [59].

Our results showed how ‘getting your life back’ does not necessarily mean the absence of pain. Although OBT-A did not completely eliminate pain, all participants perceived a very positive significant change in their lives. They acknowledged that, thanks to the treatment, they regained their life to a greater or lesser extent, regained their ‘ability to do things’, became less dependent on medication and resumed their work and/or studies; in short, they returned to a ‘normal life’. The same perceptions were reported in the study by Wilderman et al. [35]. Also, The REPOSE study [55,62,63] showed how patients who received treatment with OBT-A over 24 months decreased their perception of problems in usual activities, mobility and self-care as measured by the EuroQol 5-Dimension Questionnaire. Moreover, in the same study all scores on the Migraine-Specific Quality-of-Life Questionnaire had significant positive changes: (1) the role-function restrictive score, which assesses limitations to the patient’s daily social and work-related activities; (2) the role-function preventive score, which assesses how migraine prevents these activities, and (3) the emotional function score, which assesses the patient’s emotions associated with migraine. The impact of treatment of CM with OBT-A should always consider migraine-related disability, the impairment of patients’ functioning or ability ‘to do’ in their family, social and work environment [64,65]. This would allow the identification of relevant changes in patients’ lives, which are not always accompanied by parallel changes in commonly used clinical parameters. Interestingly, although responders are generally considered to be patients in whom the number of headache days is reduced by more than 50%, headache intensity has been found to be as important as headache frequency in terms of the impact of OBT-A treatment on migraine-related disability [66].

In the present study, most patients also reported that OBT-A treatment had enabled them to reduce their consumption of symptomatic migraine drugs (such as analgesics and triptans) and had made episodes more responsive to these drugs when needed. This is consistent with the results of the qualitative study by Wilderman et al. [35] and with other real-life studies [20]. In addition, our patients had fewer visits to an emergency department for help. This is also in line with previous studies, which have shown that CM patients treated with OBT-A visit an emergency department less frequently and have fewer hospitalizations [67,68]. Beyond patients’ personal experiences, the reduced use of health care resources contributes to the cost-effectiveness of OBT-A treatment [69,70].

The main barrier perceived by participants to receiving OBT-A treatment was the management and organization of appointments for OBT-A administration. The REPOSE study [55] reported that the treatment interval was the most common deviation observed, taking more than 13 weeks between doses for 79% of the patients (*n* = 501), and more than 16 weeks for 46% (*n* = 291) of them. The REPOSE study hypothesized that this may be partly attributed to difficulties in scheduling repeated appointments. Participants also mentioned that the COVID-19 pandemic had a negative impact on the regular administration of OBT-A and its follow-up. In the survey conducted by Smith et al. during the COVID-19 pandemic [71], patients with migraine reported running out of medication more frequently than those with other diagnoses and avoided seeking medical help for new health problems because of the pandemic more than others. Al-Hashel and Ismail [72] in their self-reported survey of the ‘real-world’ impact of the COVID-19 pandemic on migraine patients found that 62% did not communicate with their neurologists, and 66% of those receiving Botox® reported a negative impact of procedure cancellation. In the same way, González-Martínez et al. [73] reported that involuntary delay of OBT-A follow-up in patients with migraine due to the COVID-19 pandemic was associated with a higher frequency of headache and migraine attacks.

Other barriers, such as possible aesthetic effects, were not considered by most of the participants in our study. In fact, only three participants described relevant aesthetic effects. In the REPOSE study [55], patients did not report safety concerns; most adverse reactions were mild or moderate as eyelid ptosis (5.4%), neck pain (2.8%), and musculoskeletal stiffness (2.7%), which were the most common. On the other hand, in our study, no patient reported the financial cost of treatment as a barrier. In contrast, Wilderman et al. [35] reported that one of the barriers that may prevent patients from deciding to undergo OBT-A treatment is economic cost. In Spain, OBT-A treatment is funded by the National Public Health System, which may explain why the cost of treatment is not seen as a barrier.

This study presents some strengths and limitations. In terms of strengths, this is the first qualitative study of OBT-A treatment for a homogeneous group of female patients with CM who received continued treatment in accordance with the PREEMPT protocol [57]. The qualitative design enables to explore and describe the participants’ perspectives in depth and helps us to understand OBT-A as a treatment for female patients with CM [74,75]. Compared to the study by Wilderman et al. on the perspective of patients with CM and the prophylactic use of OBT-A [35], our study added these new findings: (a) Some of the reasons for trying OBT-A treatment were the side effects of previously prescribed preventives and a misuse of symptomatic medications; (b) Trust, support, taking time to answer patients’ questions and truthful information from the neurologist were essential for patients’ acceptance of OBT-A treatment; (c) Patients had high expectations of OBT-A, and did not accept well that there was a delay in the onset of effects after the first injections; (d) While waiting for the effect, patients experienced different emotions (hope-frustration); (e) Patients assumed the discomfort of the first days after the injections to achieve the expected beneficial effects; (f) Treatment resulted in fewer visits to the emergency department for help with pain control, and (g) The main barrier identified was the management of appointments for OBT-A administration, while possible aesthetic effects or the cost of treatment were not perceived as relevant barriers. The explanation for the differences with the work of Wilderman et al. could be partly due to the fact that these authors included three different groups of patients: patients who received continued OBT-A treatment (*n* = 10); patients who discontinued OBT-A treatment (*n* = 7), and patients who were recommended for OBT-A treatment but did not proceed (*n* = 5). Moreover, our study was conducted in a different social and health care setting.

Regarding limitations, our results cannot be extrapolated to all CM patients who receive OBT-A by virtue of the qualitative study design [74]. As for the adequacy of sample size, its justification in qualitative health research is limited, and defining sample size *a priori* is problematic in the case of exploratory qualitative research [75,76]. Still, we opted for Turner-Bowker’s proposal to establish a starting point for an *a priori* sample size based on empirical criteria in order to obtain the maximum percentage of narrative content from participants [46]. On the other hand, a longitudinal evaluation reflecting the patients’ point of view before and after treatment would have allowed a better understanding of certain aspects, as experience with therapies may change some perceptions. In the same line, the responders and non-responders could have different perceptions on OBT-A treatment which could be further analyzed in future research. Finally, some socio-demographic data of the participants, such as level of education and employment, were not recorded in the present study; therefore, we could not take these factors into account.

## Conclusions

Women with CM who received OBT-A in this cohort rate the treatment positively, despite the delay in prescription, discomfort during administration and some difficulties in following the treatment. All participants agree that they have regained their lives to a greater or lesser extent. Our results can help in the clinical setting to avoid delays in prescribing OBT-A treatment, to provide accurate information to patients, to create realistic expectations, and to manage and schedule successive treatment administrations. In the future, it would be necessary to keep studying patients’ experience regarding OBT-A and other therapies for CM to better understand their responses to treatment.

## Supplementary Material

Supplemental Material

## Data Availability

The data that support the findings of the study are available on request from the corresponding author, upon reasonable request. The data are not public due to ethics restrictions.

## References

[1] Headache Classification Committee of the International Headache Society (IHS). The international classification of headache disorders, 3rd edition. Cephalalgia. 2018;38(1):1–14. doi: 10.1177/0333102417738202. 29368949

[2] Kung D, Rodriguez G, Evans R. Chronic migraine: diagnosis and management. Neurol Clin. 2023;41(1):141–159. doi: 10.1016/j.ncl.2022.05.005. 36400552

[3] Buse DC, Greisman JD, Baigi K, et al. Migraine progression: a systematic review. Headache. 2019;59(3):306–338. doi: 10.1111/head.13459. 30589090

[4] Bigal ME, Lipton RB. Concepts and mechanisms of migraine chronification. Headache. 2008;48(1):7–15. doi: 10.1111/j.1526-4610.2007.00969.x. 18184280

[5] Xu J, Kong F, Buse DC. Predictors of episodic migraine transformation to chronic migraine: a systematic review and meta-analysis of observational cohort studies. Cephalalgia. 2020;40(5):503–516. doi: 10.1177/0333102419883355. 31635478

[6] Natoli JL, Manack A, Dean B, et al. Global prevalence of chronic migraine: a systematic review. Cephalalgia. 2010;30(5):599–609. doi: 10.1111/j.1468-2982.2009.01941.x. 19614702

[7] Lipton RB, Manack Adams A, Buse DC, et al. A comparison of the chronic migraine epidemiology and outcomes (CaMEO) study and American migraine prevalence and prevention (AMPP) study: demographics and headache-related disability. Headache. 2016;56(8):1280–1289. doi: 10.1111/head.12878. 27349336 PMC5132024

[8] Meletiche DM, Lofland JH, Young WB. Quality-of-life differences between patients with episodic and transformed migraine. Headache. 2001;41(6):573–578. doi: 10.1046/j.1526-4610.2001.041006573.x. 11437893

[9] Bigal ME, Rapoport AM, Lipton RB, et al. Assessment of migraine disability using the migraine disability assessment (MIDAS) questionnaire: a comparison of chronic migraine with episodic migraine. Headache. 2003;43(4):336–342. doi: 10.1046/j.1526-4610.2003.03068.x. 12656704

[10] Blumenfeld AM, Varon SF, Wilcox TK, et al. Disability, HRQoL and resource use among chronic and episodic migraineurs: results from the international burden of migraine study (IBMS). Cephalalgia. 2011;31(3):301–315. doi: 10.1177/0333102410381145. 20813784

[11] Paschoal JK, Lin J, Pinho RS, et al. Psychiatric symptoms may contribute to poor quality of life in adolescents with migraine. Pediatr Int. 2013;55(6):741–747. doi: 10.1111/ped.12178. 23829487

[12] Silberstein SD, Lipton RB, Dodick DW, et al. Efficacy and safety of topiramate for the treatment of chronic migraine: a randomized, double-blind, placebo-controlled trial. Headache. 2007;47(2):170–180. doi: 10.1111/j.1526-4610.2006.00684.x. 17300356

[13] Diener HC, Bussone G, Van Oene JC, et al. Topiramate reduces headache days in chronic migraine: a randomized, double-blind, placebo-controlled study. Cephalalgia. 2007;27(7):814–823. doi: 10.1111/j.1468-2982.2007.01326.x. 17441971

[14] Aurora SK, Dodick DW, Turkel CC, et al. OnabotulinumtoxinA for treatment of chronic migraine: results from the double-blind, randomized, placebo-controlled phase of the PREEMPT 1 trial. Cephalalgia. 2010;30(7):793–803. doi: 10.1177/0333102410364676. 20647170

[15] Diener HC, Dodick DW, Aurora SK, et al. OnabotulinumtoxinA for treatment of chronic migraine: results from the double-blind, randomized, placebo-controlled phase of the PREEMPT 2 trial. Cephalalgia. 2010;30(7):804–814. doi: 10.1177/0333102410364677. 20647171

[16] Silberstein SD, Dodick DW, Bigal ME, et al. Fremanezumab for the preventive treatment of chronic migraine. N Engl J Med. 2017;377(22):2113–2122. doi: 10.1056/NEJMoa1709038. 29171818

[17] Tepper S, Ashina M, Reuter U, et al. Safety and efficacy of erenumab for preventive treatment of chronic migraine: a randomised, double-blind, placebo-controlled phase 2 trial. Lancet Neurol. 2017;16(6):425–434. doi: 10.1016/S1474-4422(17)30083-2. 28460892

[18] Detke HC, Goadsby PJ, Wang S, et al. Galcanezumab in chronic migraine: the randomized, double-blind, placebo-controlled REGAIN study. Neurology. 2018;91(24):e2211–e2221. doi: 10.1212/WNL.0000000000006640. 30446596 PMC6329331

[19] Lipton RB, Goadsby PJ, Smith J, et al. Efficacy and safety of eptinezumab in patients with chronic migraine: PROMISE-2. Neurology. 2020;94(13):e1365–e1377. doi: 10.1212/WNL.0000000000009169. 32209650 PMC7274916

[20] Lanteri-Minet M, Ducros A, Francois C, et al. Effectiveness of onabotulinumtoxinA (BOTOX®) for the preventive treatment of chronic migraine: a meta-analysis on 10 years of real-world data. Cephalalgia. 2022;42(14):1543–1564. doi: 10.1177/03331024221123058. 36081276 PMC9693763

[21] Blumenfeld AM, Stark RJ, Freeman MC, et al. Long-term study of the efficacy and safety of OnabotulinumtoxinA for the prevention of chronic migraine: COMPEL study. J Headache Pain. 2018;19(1):13. doi: 10.1186/s10194-018-0840-8. 29404713 PMC5799088

[22] Rothrock JF, Adams AM, Lipton RB, et al. FORWARD study: evaluating the comparative effectiveness of OnabotulinumtoxinA and topiramate for headache prevention in adults with chronic migraine. Headache. 2019;59(10):1700–1713. doi: 10.1111/head.13653. 31559634 PMC6899480

[23] Altamura C, Brunelli N, Viticchi G, et al. Quantitative and qualitative pain evaluation in response to OnabotulinumtoxinA for chronic migraine: an observational real-life study. Toxins. 2023;15(4):284. doi: 10.3390/toxins15040284. 37104222 PMC10145239

[24] US Food and Drug Administration (FDA). Developing and submitting proposed draft guidance related to patient experience data, guidance for industry and other stakeholders. 2018. https://www.fda.gov/media/119542/download.

[25] Okun S. The missing reality of real life in real-world evidence. Clin Pharmacol Ther. 2019;106(1):136–138. doi: 10.1002/cpt.1465. 31002396 PMC6617711

[26] Creswell JW, Poth CN. Qualitative inquiry and research design. Choosing among five approaches. 4th ed. Thousand Oaks (CA): SAGE; 2018.

[27] Korstjens I, Moser A. Series: Practical guidance to qualitative research. Part 2: context, research questions and designs. Eur J Gen Pract. 2017;23(1):274–279. doi: 10.1080/13814788.2017.1375090. 29185826 PMC8816399

[28] Jacinto J, Lysandropoulos A, Leclerc M, et al. Experiences of patients with poststroke spasticity throughout a botulinum toxin treatment cycle: results from a prospective ethnographic study. Front Neurol. 2022;13:946500. doi: 10.3389/fneur.2022.946500. 36119669 PMC9479670

[29] Kerstens HCJW, Satink T, Nijkrake MJ, et al. Experienced consequences of spasticity and effects of botulinum toxin injections: a qualitative study amongst patients with disabling spasticity after stroke. Disabil Rehabil. 2021;43(25):3688–3695. doi: 10.1080/09638288.2020.1746843. 32255361

[30] Arai S, Fukase Y, Okii A, et al. Selection process for botulinum toxin injections in patients with chronic-stage hemiplegic stroke: a qualitative study. BMC Med Inform Decis Mak. 2019;19(1):280. doi: 10.1186/s12911-019-1003-9. 31856809 PMC6923967

[31] Nguyen L, Di Rezze B, Mesterman R, et al. Effects of botulinum toxin treatment in nonambulatory children and adolescents with cerebral palsy: understanding parents’ perspectives. J Child Neurol. 2018;33(11):724–733. doi: 10.1177/0883073818786567. 30039731

[32] Karadag-Saygi E, Kenis-Coskun Ö, Unalan PC, et al. Pros and cons of botulinum toxin injection therapy in cerebral palsy: a qualitative study exploring caregivers’ perspective. Child Care Health Dev. 2022;48(1):150–158. doi: 10.1111/cch.12915. 34623695

[33] Page AD, Elhayek N, Baylor C, et al. Exploring the psychosocial impact of botulinum toxin type a injections for individuals with oromandibular dystonia: a qualitative study of patients’ experiences. Am J Speech Lang Pathol. 2021;30(3S):1314–1328. doi: 10.1044/2020_AJSLP-20-00124. 33647215

[34] Baylor CR, Yorkston KM, Eadie TL, et al. The psychosocial consequences of BOTOX injections for spasmodic dysphonia: a qualitative study of patients’ experiences. J Voice. 2007;21(2):231–247. doi: 10.1016/j.jvoice.2006.01.007. 16564675 PMC2649951

[35] Wilderman I, Tallarigo D, Pugacheva-Zingerman O. A qualitative study to explore patient perspectives of prophylactic treatment with OnabotulinumtoxinA for chronic migraine. Pain Ther. 2021;10(2):1523–1536. doi: 10.1007/s40122-021-00316-2. 34523107 PMC8586057

[36] Jack SM, Phoenix M. Qualitative health research in the fields of developmental medicine and child neurology. Dev Med Child Neurol. 2022;64(7):830–839. doi: 10.1111/dmcn.15182. 35156198

[37] Bradshaw C, Atkinson S, Doody O. Employing a qualitative description approach in health care research. Glob Qual Nurs Res. 2017;4(4):2333393617742282. doi: 10.1177/2333393617742282. 29204457 PMC5703087

[38] Colorafi KJ, Evans B. Qualitative descriptive methods in health science research. HERD. 2016;9(4):16–25. doi: 10.1177/1937586715614171. PMC7586301 26791375

[39] Sandelowski M. What’s in a name? Qualitative description revisited. Res Nurs Health. 2010;33(1):77–84. doi: 10.1002/nur.20362. 20014004

[40] O’Brien BC, Harris IB, Beckman TJ, et al. Standards for reporting qualitative research: a synthesis of recommendations. Acad Med. 2014;89(9):1245–1251. doi: 10.1097/ACM.0000000000000388. 24979285

[41] Tong A, Sainsbury P, Craig J. Consolidated criteria for reporting qualitative research (COREQ): a 32-item checklist for interviews and focus groups. Int J Qual Health Care. 2007;19(6):349–357. doi: 10.1093/intqhc/mzm042. 17872937

[42] Sandelowski M, Barroso J. Classifying the findings in qualitative studies. Qual Health Res. 2003;13(7):905–923. doi: 10.1177/1049732303253488. 14502957

[43] Sandelowski M. Whatever happened to qualitative description? Res Nurs Health. 2000;23(4):334–340. doi: . 10940958 10.1002/1098-240x(200008)23:4<334::aid-nur9>3.0.co;2-g

[44] Moser A, Korstjens I. Series: Practical guidance to qualitative research. Part 3: sampling, data collection and analysis. Eur J Gen Pract. 2018;24(1):9–18. doi: 10.1080/13814788.2017.1375091. 29199486 PMC5774281

[45] Blumenfeld AM, Silberstein SD, Dodick DW, et al. Insights into the functional anatomy behind the preempt injection paradigm: guidance on achieving optimal outcomes. Headache. 2017;57(5):766–777. doi: 10.1111/head.13074. 28387038 PMC5434948

[46] Turner-Bowker DM, Lamoureux RE, Stokes J, et al. Informing a priori sample size estimation in qualitative concept elicitation interview studies for clinical outcome assessment instrument development. Value Health. 2018;21(7):839–842. doi: 10.1016/j.jval.2017.11.014. 30005756

[47] Paulus TM, Lester JN. Doing qualitative research in a digital world. Sage, Thousand, Oaks; 2022.

[48] Miles MB, Huberman AM, Saldaña J. Qualitative data analysis. A methods sourcebook. 3rd ed. Thousand Oaks (CA): Sage; 2014.

[49] Nowell LS, Norris JM, White DE, et al. Thematic analysis: striving to meet the trustworthiness criteria. Int J Qual Methods. 2017;16(1):160940691773384. doi: 10.1177/1609406917733847.

[50] Korstjens I, Moser A. Series: practical guidance to qualitative research. Part 4: trustworthiness and publishing. Eur J Gen Pract. 2018;24(1):120–124. doi: 10.1080/13814788.2017.1375092. 29202616 PMC8816392

[51] Palacios-Ceña D, Neira-Martín B, Silva-Hernández L, et al. Living with chronic migraine: a qualitative study on female patients’ perspectives from a specialised headache clinic in Spain. BMJ Open. 2017;7(8):e017851. doi: 10.1136/bmjopen-2017-017851. PMC5724120 28827275

[52] Minen MT, Anglin C, Boubour A, et al. Meta-Synthesis on migraine management. Headache. 2018;58(1):22–44. doi: 10.1111/head.13212. 29159874

[53] Peters M, Abu-Saad HH, Vydelingum V, et al. Patients’ decision-making for migraine and chronic daily headache management. A qualitative study. Cephalalgia. 2003;23(8):833–841. doi: 10.1046/j.1468-2982.2003.00590.x. 14510931

[54] Peters M, Huijer Abu-Saad H, Vydelingum V, et al. The patients’ perceptions of migraine and chronic daily headache: a qualitative study. J Headache Pain. 2005;6(1):40–47. doi: 10.1007/s10194-005-0144-7. 16362190 PMC3451956

[55] Ahmed F, Gaul C, García-Moncó JC, et al. An open-label prospective study of the real-life use of onabotulinumtoxinA for the treatment of chronic migraine: the REPOSE study. J Headache Pain. 2019;20(1):26. doi: 10.1186/s10194-019-0976-1. 30845917 PMC6734221

[56] Driessen MT, Cohen JM, Thompson SF, et al. Real-world effectiveness after initiating fremanezumab treatment in US patients with episodic and chronic migraine or difficult-to-treat migraine. J Headache Pain. 2022;23(1):56. doi: 10.1186/s10194-022-01415-x. 35578182 PMC9109352

[57] Bendtsen L, Sacco S, Ashina M, et al. Guideline on the use of onabotulinumtoxinA in chronic migraine: a consensus statement from the european headache federation. J Headache Pain. 2018;19(1):91. doi: 10.1186/s10194-018-0921-8. 30259200 PMC6755553

[58] Domínguez C, Pozo-Rosich P, Torres-Ferrús M, et al. OnabotulinumtoxinA in chronic migraine: predictors of response. A prospective multicentre descriptive study. Eur J Neurol. 2018;25(2):411–416. doi: 10.1111/ene.13523. 29171146

[59] Gago-Veiga AB, Santos-Lasaosa S, Cuadrado ML, et al. Evidence and experience with onabotulinumtoxinA in chronic migraine: recommendations for daily clinical practice. Neurologia. 2019;34(6):408–417. doi: 10.1016/j.nrl.2017.09.008. 29169810

[60] Cuadrado Perez ML, López Bravo A, Casas Limón J, et al. I. Bloqueos anestésicos. Infiltración con onabotulinumtoxina. In: Santos Lasaosa S, Pozos Rosich P (eds) Manual de práctica clínica en cefaleas. Sociedad española de neurologia., Madrid: Luzan 5; 2020. https://www.sen.es/pdf/2020/ManualCefaleas2020.pdf

[61] Silberstein SD, Dodick DW, Aurora SK, et al. Per cent of patients with chronic migraine who responded per onabotulinumtoxinA treatment cycle: PREEMPT. J Neurol Neurosurg Psychiatry. 2015;86(9):996–1001. doi: 10.1136/jnnp-2013-307149. 25500317 PMC4552933

[62] Ornello R, Baraldi C, Ahmed F, et al. Excellent response to OnabotulinumtoxinA: different definitions, different predictors. Int J Environ Res Public Health. 2022;19(17):10975. doi: 10.3390/ijerph191710975. 36078699 PMC9518492

[63] Alpuente A, Gallardo VJ, Torres-Ferrús M, et al. Short and mid-term predictors of response to OnabotulinumtoxinA: real-life experience observational study. Headache. 2020;60(4):677–685. doi: 10.1111/head.13765. 32086801

[64] Dekker F, Knuistingh Neven A, Andriesse B, et al. Prophylactic treatment of migraine; the patient’s view, a qualitative study. BMC Fam Pract. 2012;13(1):13. doi: 10.1186/1471-2296-13-13. 22405186 PMC3359207

[65] Leonardi M, Raggi A. A narrative review on the burden of migraine: when the burden is the impact on people’s life. J Headache Pain. 2019;20(1):41. doi: 10.1186/s10194-019-0993-0. 31023226 PMC6734273

[66] Torres-Ferrus M, Gallardo VJ, Alpuente A, et al. Influence of headache pain intensity and frequency on migraine-related disability in chronic migraine patients treated with OnabotulinumtoxinA. J Headache Pain. 2020;21(1):88. doi: 10.1186/s10194-020-01157-8. 32652924 PMC7353810

[67] Hepp Z, Rosen NL, Gillard PG, et al. Comparative effectiveness of onabotulinumtoxinA versus oral migraine prophylactic medications on headache-related resource utilization in the management of chronic migraine: retrospective analysis of a US-based insurance claims database. Cephalalgia. 2016;36(9):862–874. doi: 10.1177/0333102415621294. 26692400

[68] Kollewe K, Gaul C, Gendolla A, et al. Real-life use of onabotulinumtoxinA reduces healthcare resource utilization in individuals with chronic migraine: the REPOSE study. J Headache Pain. 2021;22(1):50. doi: 10.1186/s10194-021-01260-4. 34078259 PMC8173963

[69] Rothrock JF, Bloudek LM, Houle TT, et al. Real-world economic impact of onabotulinumtoxinA in patients with chronic migraine. Headache. 2014;54(10):1565–1573. doi: 10.1111/head.12456. 25298117 PMC4282490

[70] Ruggeri M. The cost effectiveness of Botox in italian patients with chronic migraine. Neurol Sci. 2014;35(Suppl 1):45–47. doi: 10.1007/s10072-014-1741-5. 24867835

[71] Smith M, Nakamoto M, Crocker J, et al. Early impact of the COVID-19 pandemic on outpatient migraine care in Hawaii: results of a quality improvement survey. Headache. 2021;61(1):149–156. doi: 10.1111/head.14030. 33316097

[72] Al-Hashel JY, Ismail II. Impact of coronavirus disease 2019 (COVID-19) pandemic on patients with migraine: a web-based survey study. J Headache Pain. 2020;21(1):115. doi: 10.1186/s10194-020-01183-6. 32972360 PMC7513457

[73] Gonzalez-Martinez A, Planchuelo-Gómez Á, Guerrero ÁL, et al. Effects of the onabotulinumtoxinA follow-up delay in migraine course during the COVID-19 lockdown. Neurol Sci. 2021;42(12):5087–5092. doi: 10.1007/s10072-021-05180-8. 33768436 PMC7994064

[74] Sebele-Mpofu FY. Saturation controversy in qualitative research: complexities and underlying assumptions. A Literature Review. Cogent Soc Sci. 2020;6(1):1838706.

[75] Vasileiou K, Barnett J, Thorpe S, et al. Characterising and justifying sample size sufficiency in interview-based studies: systematic analysis of qualitative health research over a 15-year period. BMC Med Res Methodol. 2018;18(1):148. doi: 10.1186/s12874-018-0594-7. 30463515 PMC6249736

[76] Sim J, Saunders B, Waterfield J, et al. Can sample size in qualitative research be determined a priori? Int J Soc Res Method. 2018;21(5):619–634. doi: 10.1080/13645579.2018.1454643.

