# Supplementary material for: Real-world experience of OnabotulinumtoxinA treatment in female patients with chronic migraine: a qualitative study using in-depth interviews
Source: Ann Med. 2023 Sep 14;55(2):2255215. doi: 10.1080/07853890.2023.2255215 (PMC10936648; doi:10.1080/07853890.2023.2255215)
Supplement: Supplemental Material [file IANN_A_2255215_SM0846.doc]

**Additional file 1****.** Sociodemographic and clinical data of each participant.

|  | Age | Time since migraine onset (years) | Time since chronic migraine onset (months) | Presence of aura | Other preventives used (in the past/currently) | Duration of Botox® treatment (months) | Last dose of Botox® (units) | Headache days per month before Botox® treatment (average) | Headache days per month in the last 3 months (average) | Moderate-to-severe headache days per month in the last 3 months (average) | Days per month with acute rescue medication in the last 3 months (average) | Side effects with Botox® treatment |
| --- | --- | --- | --- | --- | --- | --- | --- | --- | --- | --- | --- | --- |
| P1 | 46 | 34 | 180 | No | Topiramate, zonisamide, propranolol, atenolol, flunarizine, amitryptiline, nortryptiline, duloxetine, venlafaxine | 79 | 195 | 24 | 10 | 10 | 10 | No |
| P2 | 41 | 25 | 192 | No | Topiramate, propranolol | 41 | 195 | 30 | 6 | 2 | 2 | No |
| P3 | 29 | 15 | 50 | Yes | Topiramate, zonisamide, propranolol, flunarizine, amitryptiline | 36 | 195 | 25 | 10 | 3 | 3 | No |
| P4 | 36 | 23 | 144 | No | Topiramate, propranolol, amitryptiline, duloxetine, gabapentin | 52 | 155 | 16 | 7 | 3 | 3 | No |
| P5 | 45 | 29 | 72 | No | Topiramate, zonisamide, nebivolol, flunarizine, amitriptyline | 65 | 195 | 23 | 2 | 2 | 2 | No |
| P6 | 28 | 10 | 93 | Yes | Topiramate, flunarizine, amitriptyline | 41 | 155 | 30 | 1 | 1 | 1 | No |
| P7 | 47 | 25 | 60 | No | Topiramate, flunarizine, amitriptyline | 50 | 195 | 17 | 4 | 4 | 4 | No |
| P8 | 37 | 36 | 96 | No | Topiramate, nebivolol, amitriptyline, nortriptyline venlafaxine | 26 | 195 | 18 | 5 | 4 | 4 | Aesthetic changes |
| P9 | 47 | 10 | 60 | No | Topiramate, propranolol, amitriptyline | 36 | 195 | 16 | 2 | 1 | 1 | No |
| P10 | 40 | 15 | 88 | No | Topiramate, amitriptyline | 72 | 155 | 30 | 2 | 0 | 2 | No |
| P11 | 41 | 29 | 252 | Yes | Topiramate, propranolol, nebivolol, flunarizine | 42 | 155 | 15 | 3 | 3 | 3 | No |
| P12 | 54 | 38 | 240 | Yes | Topiramate, nebivolol, amitriptyline, flunarizine | 84 | 195 | 15 | 0 | 0 | 0 | Aesthetic changes |
| P13 | 25 | 10 | 48 | No | Topiramate, amitriptyline, flunarizine | 36 | 195 | 16 | 8 | 5 | 8 | No |
| P14 | 64 | 25 | 60 | No | Topiramate, zonisamide, nebivolol, amitriptyline, flunarizine | 5 | 195 | 19 | 6 | 6 | 6 | No |
| P15 | 45 | 23 | 72 | No | Topiramate, zonisamide, propranolol, amitriptyline | 60 | 195 | 15 | 2 | 2 | 2 | No |
| P16 | 42 | 20 | 96 | Yes | Topiramate, nebivolol amitriptyline, flunarizine | 7 | 195 | 20 | 15 | 3 | 10 | No |
| P17 | 57 | 43 | 42 | No | Topiramate, nortriptyline, flunarizine | 36 | 155 | 30 | 0 | 0 | 0 | Aesthetic changes |
| P18 | 23 | 12 | 36 | No | Topiramate, zonisamide propranolol, flunarizine | 22 | 195 | 16 | 6 | 4 | 4 | No |
| P19 | 50 | 35 | 66 | No | Topiramate, propranolol, nebivolol, amitriptyline, flunarizine | 48 | 180 | 19 | 5 | 5 | 5 | No |
| P20 | 63 | 42 | 225 | No | Topiramate, valproate, amitriptyline, venlafaxine, duloxetine, mirtazapine, flunarizine | 147 | 155 | 15 | 6 | 0 | 0 | No |
| P21 | 46 | 24 | 60 | No | Topiramate, propranolol, amitriptyline, flunarizine | 40 | 190 | 16 | 3 | 3 | 3 | No |
| P22 | 46 | 22 | 85 | Yes | Topiramate, zonisamide propranolol, amitriptyline, flunarizine | 26 | 195 | 22 | 7 | 5 | 5 | No |
| P23 | 45 | 16 | 65 | No | Topiramate, valproate, amitriptyline, flunarizine, chlorpromazine | 48 | 195 | 23 | 10 | 8 | 10 | No |
| P24 | 23 | 16 | 80 | No | Topiramate, propranolol, amitriptyline, flunarizine | 25 | 195 | 16 | 12 | 6 | 8 | No |
| P25 | 42 | 19 | 110 | No | Topiramate, propranolol, amitriptyline, venlafaxine, duloxetine | 88 | 185 | 28 | 10 | 5 | 7 | No |
| P26 | 37 | 11 | 98 | No | Topiramate, amitriptyline | 76 | 195 | 28 | 5 | 1 | 1 | No |
| P27 | 55 | 31 | 38 | No | Topiramate, zonisamide, valproate, amitriptyline | 30 | 165 | 17 | 0 | 0 | 0 | No |
| P28 | 34 | 12 | 85 | Yes | Topiramate, nebivolol, amitriptyline, | 32 | 195 | 18 | 12 | 6 | 8 | No |
| P29 | 43 | 28 | 89 | No | Topiramate, amitriptyline, flunarizine | 52 | 155 | 24 | 1 | 1 | 1 | No |
| P30 | 50 | 36 | 276 | Yes | Topiramate, amitriptyline, flunarizine, duloxetine, pregabalin, gabapentin | 85 | 195 | 30 | 24 | 2 | 5 | No |
